# Shikonin exerts antitumor activity in Burkitt’s lymphoma by inhibiting C-MYC and PI3K/AKT/mTOR pathway and acts synergistically with doxorubicin

**DOI:** 10.1038/s41598-018-21570-z

**Published:** 2018-02-20

**Authors:** Fan Ni, Xianbo Huang, Zhenzhen Chen, Wenbin Qian, Xiangmin Tong

**Affiliations:** 1 The Second Clinical Medical College, Zhejiang Chinese Medical University, Hangzhou, Zhejiang, 310053 P.R. China; 2 Clinical Research Institute, Zhejiang Provincial People’s Hospital, Hangzhou, Zhejiang, 310014 P.R. China; 3 Key Laboratory of Cancer Molecular Diagnosis and Individualized Therapy of Zhejiang Province, Hangzhou, Zhejiang, 310014 P.R. China; 4 Malignant Lymphoma Diagnosis and Therapy Center, the First Affiliated Hospital, College of Medicine, Zhejiang University, Hangzhou, 310003 P.R. China

## Abstract

Burkitt’s lymphoma (BL) is a highly aggressive malignancy molecularly characterized by deregulation of the C-MYC proto-oncogene. Recently, it has been confirmed that phosphatidylinositol-3-kinase (PI3K) pathway activation is a crucial element in the malignant transformation of the B cells in BL. Despite the better outcome of adults with BL treated with high-intensity chemotherapy regimens, the overall survival rate for patients older than 60 years remains dismal. Shikonin, a natural naphthoquinone derived from Chinese herbal medicine plant, has the potential to induce cell death in a series of human cancer. In the present study, we investigated the effect and molecular mechanisms of Shikonin in treatment with BL. Shikonin suppressed cellular proliferation and induced caspase-dependent apoptosis in BL cells. Inhibition of C-MYC and suppression of PI3K/AKT/mTOR pathway played critical roles in SHK-induced apoptosis in BL both *in vitro* and *in vivo*. Besides, Shikonin potentiated doxorubicin-induced growth inhibition and apoptosis *in vitro*. Furthermore, the growth of a subcutaneous xenograft tumor model of BL was significantly inhibited by shikonin. Importantly, we did not find the effect of shikonin on liver function in mice. In summary, these data suggest that shikonin may be an encouraging chemotherapeutic agent in the clinical treatment of BL.

## Introduction

Burkitt’s lymphoma (BL) is a highly aggressive malignancy derived from germinal center B cells, with a cellular doubling time of 25 hours^1^. It is molecularly characterized by deregulation of the C-MYC proto-oncogene as a result of the juxtaposition of C-MYC to the enhancer elements of one of the immunoglobulin genes: the heavy chain, the kappa light chain and the lambda light chain. During the past decades, the outcome of adults with BL treated with high-intensity chemotherapy regimens has improved substantially, with overall survival rates exceeding 70%^1,2^. However, survival for patients older than 60 years remains dismal^3,4^. And the main reason for the poor outcome of the elderly with BL may be intolerance of intensive chemotherapies. Therefore, novel therapeutic strategies are still required to obtain less toxic side effects but more effective activities for the elderly with BL.

Substantial evidence has proved that C-MYC deregulation has a range of implications in cancer-associated events that include aberrant cell proliferation, metabolic reprogramming, and genomic instability. In fact, C-MYC translocation and overexpression are observed in all BL cases^5^. However, the overexpression of C-MYC alone is not sufficient for Burkitt lymphomagenesis considering its association with apoptotic effects^6^. Recently, two independent studies provided fundamental insights into the pathogenesis of BL that phosphatidylinositol-3-kinase (PI3K) pathway activation is a crucial element in the malignant transformation of the B cells^7,8^. In BL cells, a constitutive “tonic” B cell receptor (BCR) signaling rather than antigen-dependent “active” BCR signaling which engages NF-κB pathway mainly mediates the activation of the PI3K pathway. Transcription factor 3 (TCF-3), a constitutively activated transcription factor also involves the activation of PI3K through augmenting the BCR signaling. Additionally, C-MYC contributes to the activation of the PI3K pathway by promoting expression of mir-17–92 cluster, one of which decreases expression of phosphatase and tensin homolog deleted on chromosome ten (PTEN)^9^. Overall, these results demonstrate that C-MYC and PI3K pathway have emerged as promising therapeutic targets in BL.

Natural herbs play a vital role in cancer treatment owing to their pharmacological effects and low toxicity^10,11^. Shikonin (SHK) is an active naphthoquinone derivative compound extracted from root tissues of the traditional Chinese medical herb *Lithospermum erythrorhizon* which had been broadly applied by ancient Chinese doctors for thousands of years to treat burns and to promote wound healing. Recent emerging researches confirmed that SHK had the potential to induce apoptosis in a variety of human tumor cell lines including leukemia cell lines *in vitro* and *in vivo* with minimal or no toxicity to healthy human cells^12–15^. The anti-tumor activity of SHK may involve in its ability to generate reactive oxygen species^12^, activate pro-apoptotic caspase family members, inhibit the expression of C-MYC^13^ and suppress PI3K phosphorylation^14^. Thus much attention has been focused on the potential value of SHK in the therapy of kinds of cancer. However, the effect of SHK on the growth of BL and the possible mechanism had never been reported.

In this study, we investigated whether SHK alone could have the anti-tumor effect on BL cells both *in vitro* and in a xenograft mouse model, and whether SHK could have the potential to act as a chemosensitizing agent to improve the therapeutic index of doxorubicin (DOX) *in vitro*. The results showed that SHK suppressed cellular proliferation and induced caspase-dependent apoptosis through inhibition of the expression of C-MYC and the modulation of PI3K signaling in BL cells. Besides, SHK strongly potentiated DOX-induced growth inhibition and apoptosis. The present data suggest that SHK may serve as a novel agent for the treatment of BL because of its likely targets.

## Results

### Shikonin suppresses cellular proliferation and induces caspase-dependent apoptosis in Burkitt’s lymphoma cells

**Figure 1:**
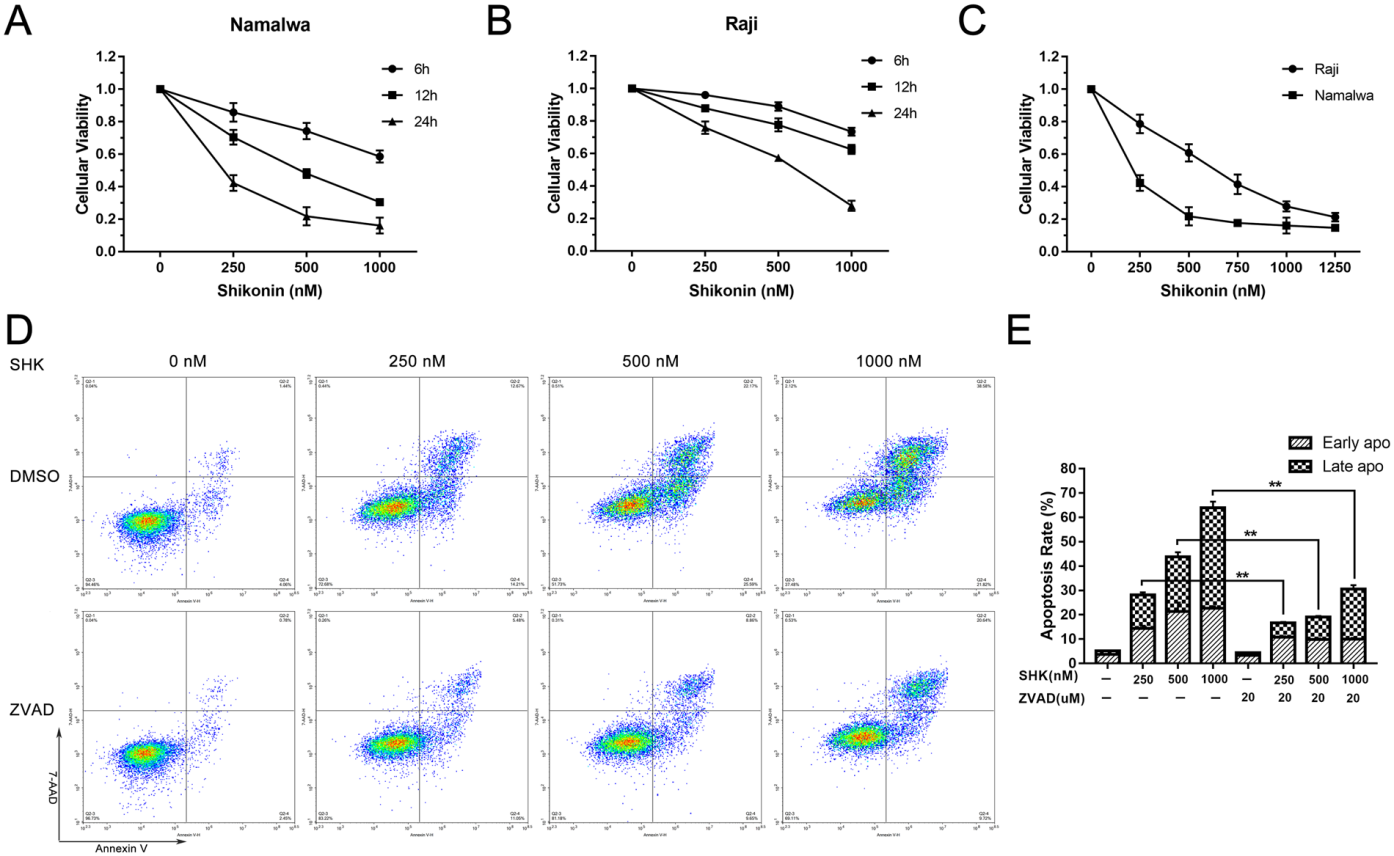
Shikonin potently inhibits cell growth in BL cell lines. (**A**) and (**B**) Namalwa and Raji cells were plated in 96-well plates and treated with SHK at indicated concentrations for different times. (**C**) Cells were treated with increasing concentrations of SHK for 24 h. The MTT assay was then used to quantify the viability of cells. (**D**) Namalwa cells were treated with increasing doses of SHK for 24 h with or without ZVAD-FMK (20 μM) and flow cytometry was performed to examine the apoptotic cells. (**E**) Percentage of apoptotic cells after treatment with indicated concentrations of SHK for 24 h. Data are presented as the mean ± SD of three independent experiments. ** represents P < 0.01.

For investigating the effect of SHK on the proliferation of human BL cells, Raji and Namalwa cells were treated with different concentration of SHK for various durations. Cell viability measured by a MTT assay showed that SHK suppresses cellular proliferation in dose-dependent and time-dependent manners (Fig. 1A–C). The two cell lines responded differently to the SHK treatment and Namalwa cells were apparently more sensitive to SHK than Raji cells. The differences noted between two cell lines were in agreement with the IC50 values (for Raji and Namalwa were 588.31 nM and 192.67 nM at 24 h, respectively) determined for each cell line exposed to SHK.

Previous researches demonstrated that induction of apoptosis is the main way for SHK-induced cell death *in vitro*^12,15^. So we detected whether apoptosis also took part in this process in BL after SHK treatment, and the apoptosis rate of Namalwa cells was examined using Annexin V and 7-AAD staining assay followed by flow cytometry analysis. As shown in Fig. 1D, the percentage of apoptotic cells dramatically increased with growing concentration of SHK from 250 to 1000 nM after incubation for 24 h. Furthermore, the pan-caspase inhibitor ZVAD-FMK (20 μM) was administered in advance for 2 h to determine whether the apoptosis induced by SHK was caspase-dependent. And we found that the apoptosis rate was significantly decreased, especially for the late apoptosis compared to the early apoptosis (Fig. 1D,E).

**Figure 2:**
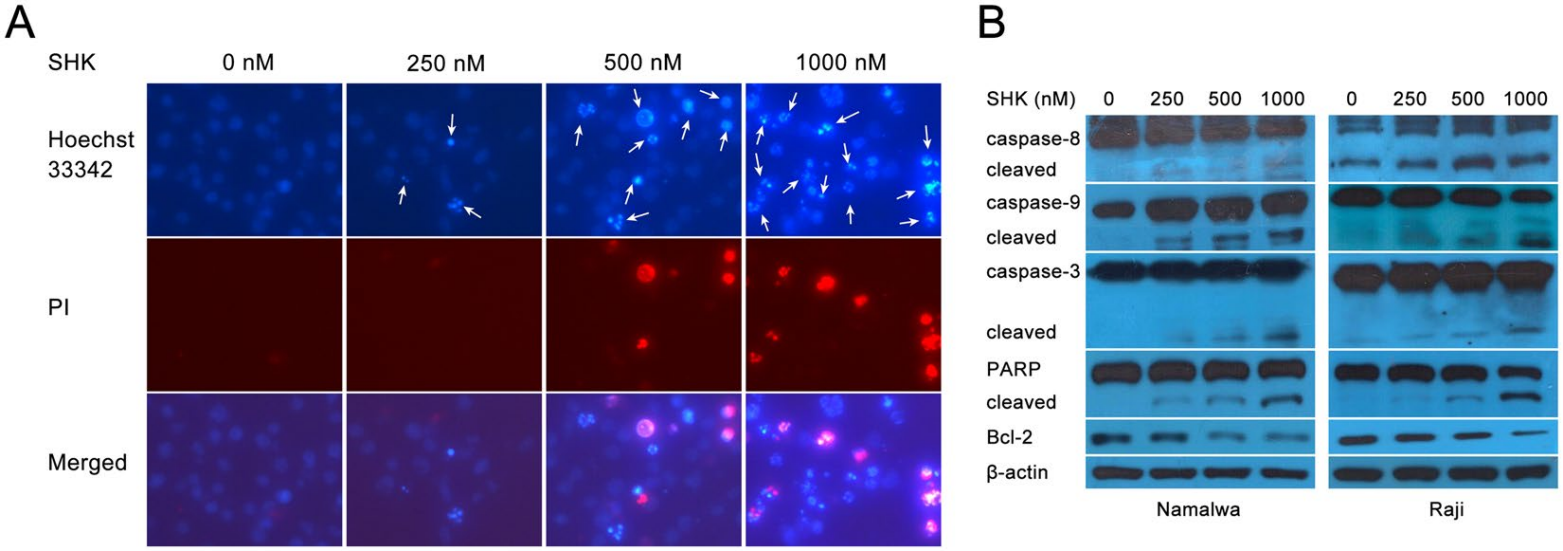
Shikonin induces apoptosis through a caspase-dependent manner in BL cell lines. (**A**) Fluorescence microscopy (100x) with Hoechst 33342 and PI dual staining showed that the nuclear condensation or fragmentation after SHK treatment for 6 h. (**B**) Namalwa and Raji cells treated with SHK in the indicated concentrations for 12 h were taken for the detection of activation of caspase pathway using western blotting analysis. β-actin was used as a loading control. All data represent an independent experiment from three repeated tests with similar results. Cropped blots/gels were used in the figure and the gels had been run under the same experimental conditions; the fulllength blots/gels are presented in Supplementary Figure S1.

To observe the nuclear condensation or fragmentation which signifies apoptosis, we used Hoechst 33342 and PI staining in Namalwa cells after 6 h incubation with SHK. As shown in Fig. 2A, on the contrast with untreated cells presenting round nuclei with dark blue color, part of the cells treated by SHK had shrunken nuclei in light blue color without or with red color indicating they were early apoptotic cells or late apoptotic cells, respectively. Furthermore, the numbers of apoptotic cells were also positively related to the concentration of SHK treatment. On western blot analysis (Fig. 2B), the same doses of SHK dose-dependently activated caspase pathway as evidenced by splicing events of caspase-9, -8, -3 and PARP, which indicate the initiation of apoptosis. All these data suggest that SHK activates caspase cascade and induces caspase-dependent apoptosis in BL cells.

### Shikonin inhibits both C-MYC and PI3K/AKT/mTOR activity ***in vitro***

**Figure 3:**
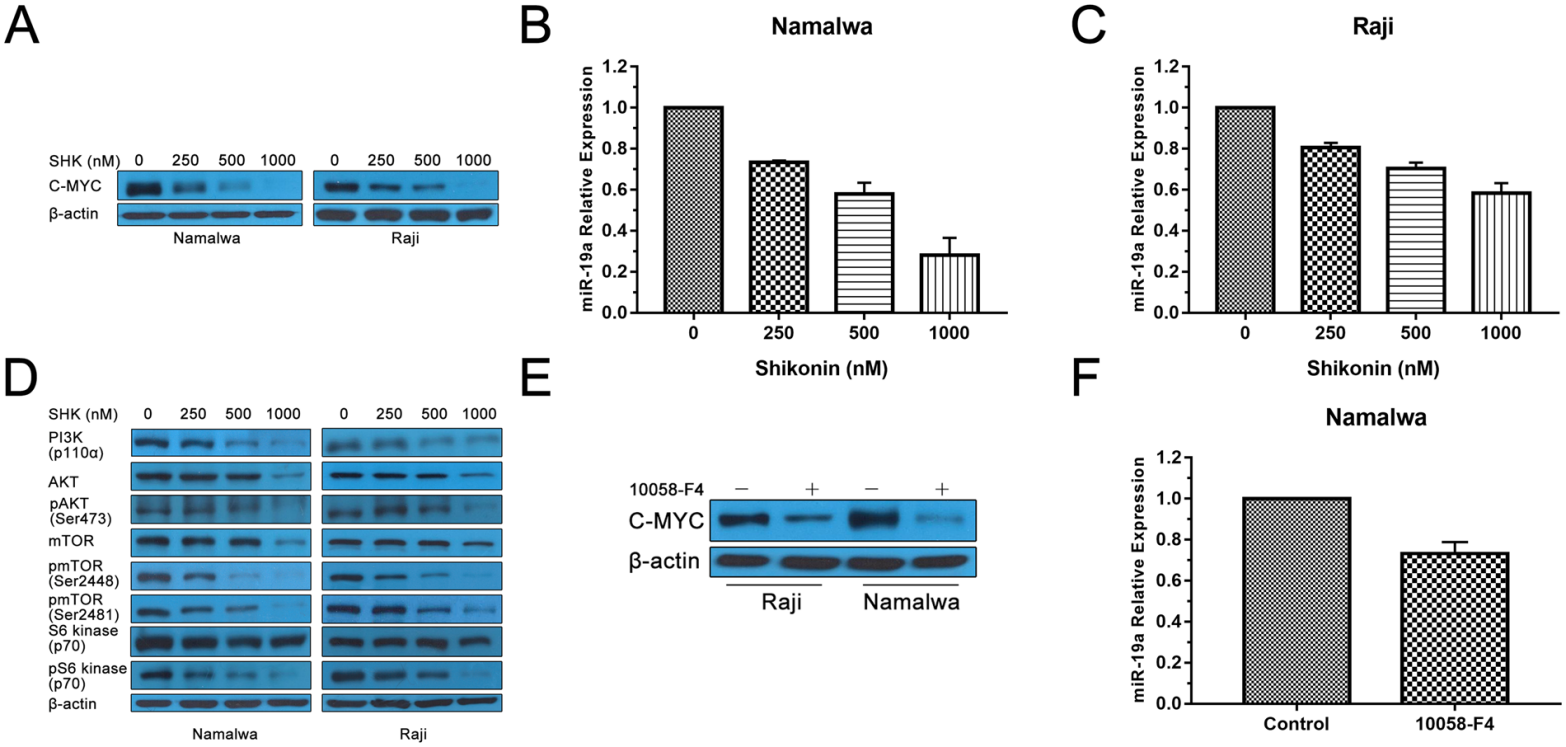
Shikonin inhibits both C-MYC and PI3K/AKT/mTOR activity *in vitro*. (**A**) Western blot indicated the protein expression of C-MYC in BL cells after treatment with SHK. β-actin was used as a loading control. (**B**) and (**C**) Namalwa and Raji cells were treated with SHK for 12 h. The relative expression of miR-19a was analyzed by RT-PCR. Data are presented as the mean ± SD of three independent experiments. (**D**) Western blot indicated the protein expression of phosphorylation of PI3K/AKT/mTOR in BL cells after treatment with SHK. β-actin was used as a loading control. (**E**) The protein expression of C-MYC was analyzed by western blot after treatment with C-MYC inhibitor 10058-F4 (100 uM) in BL cells. β-actin was used as a loading control. Cropped blots/gels were used in the figure and the gels had been run under the same experimental conditions; the fulllength blots/gels are presented in Supplementary Figure S2. (**F**) RT-PCR was performed to evaluate the effect of 10058-F4 (100 uM) on expression of miR-19a in Namalwa cells. All data represent an independent experiment from three repeated tests with similar results.

Numerous studies have established that C-MYC deregulation is one of the most important events for BL malignant transformation^16,17^. The previous study showed that C-MYC is a potential target of SHK in U937 cells^13^. Therefore, we evaluated the effect of SHK on C-MYC in Namalwa and Raji cells. As showed in Fig. 3A, SHK significantly inhibited the protein expression of C-MYC in a dose-dependent manner in both BL cells.

C-MYC regulates a large number of micro-RNAs (miRs) that function as oncogenes apart from inducing cell proliferation and growth^18^. MiR-19a is one of the oncogenic miR-17-92-cluster members which is up-regulated by C-MYC and can cause the activation of the PI3K/AKT pathway through down-regulation of PTEN^9^. In this study, we found that SHK also induced a dose-dependent reduction of miR-19a for Namalwa cells (Fig. 3B). Similar results were obtained with Raji cells (Fig. 3C). A known C-MYC inhibitor 10058-F4 was used as a positive control drug. As shown in Fig. 3E,F, 10058-F4 inhibited the expression of both C-MYC and miR-19a.

It is known that PI3K activity plays a central role in the development and ongoing maintenance of BL^19,20^. And several studies found that SHK could inhibit PI3K activity in different tumor cells^14,21^. So we first investigated the role of SHK on PI3K and its downstream AKT expression by western blot analysis (Fig. 3D), SHK decreased p110α which is one of the PI3K subunits and phosphorylation of AKT at Ser473 in a dose-dependent fashion in Namalwa and Raji cells. As expected, SHK also strongly inhibited phosphorylation of mTOR at Ser2448, a marker for mTORC1 activity^22^, as well as phosphorylation of p70S6K, the best-characterized target of mTORC1. Moreover, SHK successfully inhibited phosphorylation of mTOR at Ser2481, a marker for the presence of mTORC2 complexes^22^, which was consistent with the decreased level of phosphorylation of AKT at Ser473. The activity of mTORC1 and mTORC2 in Namalwa and Raji cells was completely inhibited by the treatment with 1000 nM SHK accompanied by slight degradation of protein expression of mTOR.

### The synergistic anti-proliferation activity of shikonin and doxorubicin is that Shikonin potentiates doxorubicin-induced apoptosis in Burkitt’s lymphoma cells

**Figure 4:**
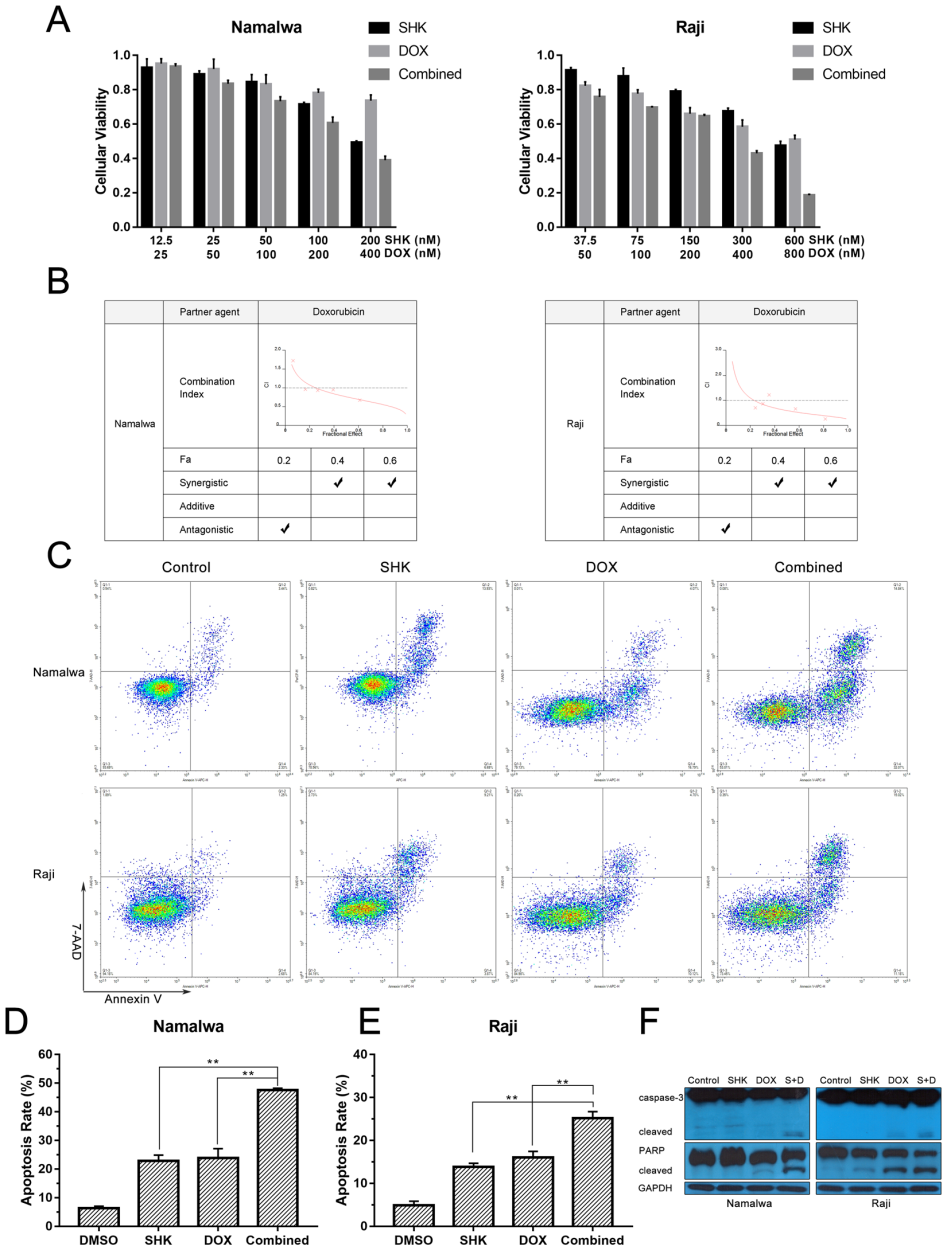
The synergistic anti-proliferation activity of shikonin and doxorubicin is that Shikonin potentiates doxorubicin-induced apoptosis in Burkitt’s lymphoma cells. (**A**) Namalwa and Raji cells were treated with a series of doses of SHK or/and DOX for 24 h, and cell viability was determined by a MTT assay. Data are presented as the mean ± SD of three independent experiments. (**B**) Combination index (CI) plots obtained from median-effect analysis of Chou-Talalay. CI values: >1, antagonism; = 1, additivity; <1, synergism. (**C**) Namalwa and Raji cells were treated with SHK (200 nM and 600 nM, respectively), DOX (400 nM and 800 nM, respectively) or in combination for 24 h. Apoptosis was analyzed by flow cytometry after dual staining of cells with Annexin V and 7-AAD. (**D**) and (**E**) Percentage of apoptotic cells after treatment with indicated drugs for 24 h. Data are presented as the mean ± SD of three independent experiments. ** represents P < 0.01. (**F**) Namalwa and Raji cells were treated with SHK (200 nM and 600 nM, respectively), DOX (400 nM and 800 nM, respectively) alone or in combination for 24 h. Cleavage of caspase-3 and PARP were analyzed by western blotting analysis. β-actin was used as a loading control. All data represent an independent experiment from three repeated tests with similar results. Cropped blots/gels were used in the figure and the gels had been run under the same experimental conditions; the fulllength blots/gels are presented in Supplementary Figure S3.

Doxorubicin is one of the most commonly used chemotherapeutic drugs in patients with BL. Given the fact that the acquisition of doxorubicin resistance is a primary cause of chemotherapy failure and high doses of doxorubicin easily lead to toxic side effects, we tested the effect of combination therapy of doxorubicin and SHK by MTT assay. Therefore, Namalwa and Raji were treated with a series of doses of SHK or/and doxorubicin. As shown in Fig. 4A, SHK (200 nM) or doxorubicin (400 nM) decreased the cell viability of Namalwa cells to 49.33% or 73.63%, respectively, whereas the viability of Namalwa cells treated with the combination therapy decreased to 38.93% and the similar result was also observed in Raji cells. The combination index analysis showed that CI range values in Namalwa cells were 1.73 to 0.68 for fractional effect corresponding to 0.06 to 0.61 (Fig. 4B), indicating that SHK in combination with doxorubicin has synergistic anti-proliferation activity.

Next, we examined whether this synergy effect of combination treatment of SHK and doxorubicin was due to induction of apoptosis. As shown in Fig. 4C–E, the results of the flow cytometric analysis revealed a marked increase in the proportion of apoptotic cells after combined treatment with SHK and doxorubicin for 24 h compared with that of SHK or doxorubicin alone. And SHK distinctly enhanced doxorubicin-induced activation of caspase-3 and PARP indicating the activation of apoptosis pathway (Fig. 4F). These data are consistent with MTT assay, suggesting that synergistic cell growth inhibition resulting from combination treatment with SHK and doxorubicin may owe to, at least partly, the induction of increased apoptosis in BL cells.

### Shikonin inhibits xenograft tumor growth

**Figure 5:**
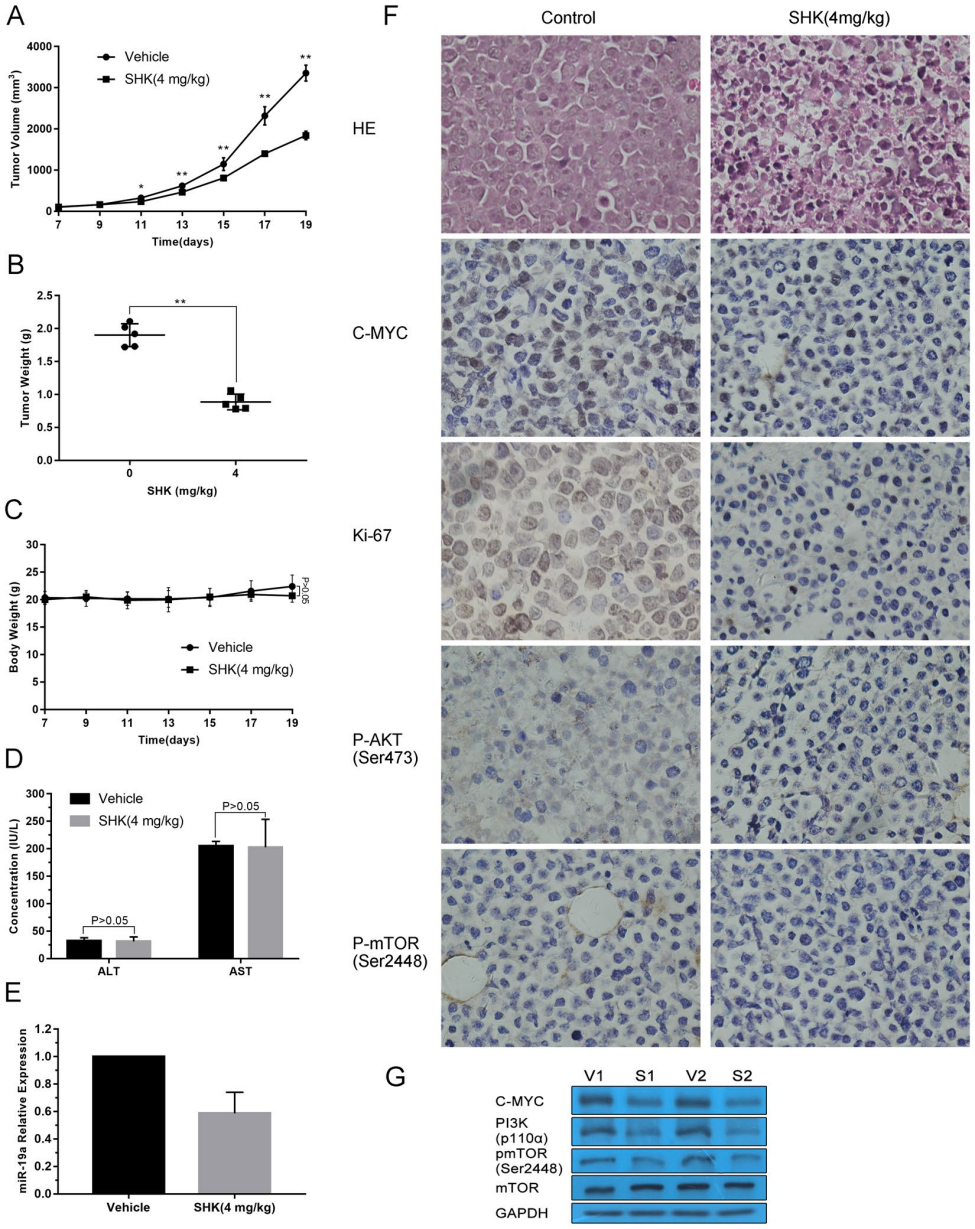
Shikonin inhibits Namalwa tumor growth *in vivo*. (**A**) Namalwa xenografts were established in mice and treated with vehicle control or SHK (4 mg/kg, i.p.). The volume of tumor was measured at the indicated times. Data are presented as mean ± SD (n = 5 per group). * and ** represents P < 0.05 and P < 0.01, respectively. (**B**) The scatter plot represents mean of the tumor weight from SHK-treated and control mice. Data are presented as mean ± SD (n = 5 per group). ** represents P < 0.01. (**C**) The body weight of mice was measured at the indicated times. Data are presented as mean ± SD (n = 5 per group). (**D**) Serum activity of alanine transaminase (ALT) and aspartate transaminase (AST) was measured in control and SHK group by spectrophotometric methods. Data are presented as mean ± SD (n = 5 per group). (**E**) RT-PCR was performed to evaluate the relative expression of miR-19a in Namalwa xenografts. Data are presented as mean ± SD (n = 3 per group). (**F**) Hematoxylin and eosin hematoxylin and eosin (HE) staining was performed to observe pathological changes in tumor tissues. Immunohistochemistry was performed to measure cell proliferation by Ki-67 staining, and the expression of C-MYC, P-AKT (Ser473) and P-mTOR (Ser2448). Original magnification: 400x. Samples from three animals in each group were analyzed, and representative data are shown. (**G**) Western blot analysis monitors the expression of C-MYC, PI3K and P-mTOR (Ser2448). Samples from two animals in each group were analyzed. GAPDH was used as a loading control. Cropped blots/gels were used in the figure and the gels had been run under the same experimental conditions; the fulllength blots/gels are presented in Supplementary Figure S4.

Given the potent inhibitory activity of SHK on BL growth *in vitro*, it is believed that SHK has potent anti-tumor effects in treating BL *in vivo*. Thus, we established NOD/SCID mice xenografts bearing Namalwa BL cells. As shown in Fig. 5A, there was a significant decrease in mean tumor volume for mice that received the intraperitoneal injection of SHK compared to which were treated with fat emulsion. The last average tumor volume of vehicle group and SHK group reached to 3356.61 mm^3^ and 1842.63 mm^3^, respectively (P < 0.01). At the end of the experiment, tumors were isolated from mice and weighted. The mean tumor weight was significantly more substantial in control group compared with SHK-treated mice (P < 0.01) (Fig. 5B). Importantly, there was no significant difference between the vehicle group and SHK group in mice mean body weight (Fig. 5C). Moreover, there was also no significant difference in serum aspartate transaminase and alanine transaminase levels between SHK-treated mice and control mice (Fig. 5D).

Additionally, consistent with our previous findings *in vitro*, the miR-19a expression of the SHK group was remarkably lower than the control group (Fig. 5E). To evaluate the histopathology changes in the tumor tissues, we performed hematoxylin and eosin (HE) and immunohistochemical staining after the tissues being extracted. As presented in Fig. 5F, HE staining demonstrated that SHK caused severe pathological changes including cell death, tumor necrosis and angiogenesis inhibition compared with control tissues. Furthermore, treatment with SHK obviously decreased the number of Ki-67-positive cells in tumors compared with controls. Similar results that SHK down-regulated C-MYC, P-AKT (Ser473) and P-mTOR (Ser2448) in xenograft tumors were observed in Fig. 5G. Collectively, our data suggest that SHK may be a safe and effective anti-tumor agent for BL, at least in xenograft tumor models. Western blot analysis showed that SHK also significantly decreased protein levels of PI3K, C-MYC and P-mTOR (Ser2448) *in vivo* (Fig. 5G).

## Discussion

With the development of intensive chemotherapy regimens, pediatric BL treatment is very successful, most of which can be cured^23^. However, the elderly patients often can not tolerate these rigorous BL regimens. Moreover, the bone marrow and the immune responses are consequently suppressed by these regimens, placing a premium on supportive care, which is to detect and treat infections promptly and efficiently. Hence, it is urgent to exploit new treatment regimens which are less immunosuppressive and better tolerated for BL.

SHK is a small molecule natural compound derived from Chinese herbal medicine plant. Since its long history used in Chinese traditional medicine, it is considered to be much safe for human beings. Recent experiments found out that SHK had anti-tumor effects on glioma^24^, gastric cancer^25^, hepatocellular carcinoma^26^ and leukemia^12,15^. In this study, we confirmed that SHK suppressed cellular proliferation and induced death in Raji and Namalwa BL cells in a dose and time-dependent manner. Apoptosis induced by chemotherapy, is one of the essential patterns participating in eliminating cancer cells. It is a consequence of a series of precisely controlled events involving cell death^27^. Two related pathways are resulting in apoptosis: the extrinsic pathway and the intrinsic pathway^28^. Generally speaking, the former involves activating caspase-8, and the latter involves activating caspase-9. Then activated caspase-8 or caspase-9 could trigger downstream proteins, such as caspase-3 and poly ADP-ribose polymerase (PARP). Our data presented that SHK induced apoptosis in BL cells via caspase-dependent pathways, which was validated by nuclear fragmentation, apoptotic cell morphology and pan-caspase inhibitor ZVAD-FMK.

C-MYC deregulation plays a critical role in the pathogenesis of BL. Indeed, BET Bromodomain inhibitors that can inhibit C-MYC indirectly have been examined in mouse models of BL and been undergoing clinical trials in patients with hematologic malignancies^29–31^. Besides chromosome translocation, some other mechanisms are accounting for C-MYC deregulation in malignancy, for instance, gene amplification and insertional viral mutagenesis^18^. The C-MYC protein usually acts as a transcription factor that binds to DNA and regulates transcription. It can make the cell phase transition from the G0/1 phase to the S phase, which ultimately induces DNA replication, protein biosynthesis and cell proliferation and growth^32^. In our study, we found that SHK could significantly decrease the expression of C-MYC in protein level even at a low dose as well as its specific inhibitor10058-F4 did at a relatively high dose in BL cells. Apart from association with cell proliferation and growth, C-MYC ties up with a great number of micro-RNAs that function as oncogenes such as miR-19a^9^ or tumor suppressor genes such as miR-34a^33^. Similar to the previous results, SHK also down-regulated the expression of miR-19a in a dose-manner in BL cells, which negatively regulated the expression of PTEN. It will be interesting to investigate the relationship between SHK and PTEN in future.

It has proved that C-MYC dysregulation alone does not lead to lymphoma^34^ and the t(8;14) is also detected in blood cells and bone marrow of healthy individuals^35^. Until recently an engineered mouse model expressing deregulated C-MYC and constitutively-active PI3K specifically in germinal center B cells identified that PI3K signaling could cooperate with C-MYC in the development of BL^7^. In the meantime, by comprehensive high-throughput RNA sequencing, Schmitz *et al*. further revealed that mutations of TCF3/ID3 genes resulted in continuous PI3K activation in a large panel of BL cases^8^. Therefore, more and more attention is paid to PI3K inhibitors and several pan-PI3K or isoform-selective PI3K inhibitors have been undergoing clinical trials in non-Hodgkin lymphoma^36,37^. In this study, we found that SHK inhibited protein expression of p110α, which is one of the catalytic subunits of Class IA PI3Ks. Moreover, SHK decreased the activity of mTORC1 and mTORC2, as evidenced by dephosphorylation of mTOR on Ser2448 and Ser2481. Paralleled with these results, it elicited potent suppression of phosphorylation of p70S6K and phosphorylation of AKT at Ser473 in BL cells, standing for one of the downstream targets of mTORC1 and a substrate of mTORC2, respectively. The data that both dephosphorylation of mTOR on Ser2448 and Ser2481 by SHK is exciting and inspiring because there is a negative feedback loop between mTORC1 and mTORC2^38^. Once mTORC1 was inhibited, mTORC2 could be further activated through PI3K signaling. It is the reason that single-agent rapamycin analogues have limited efficacy in cancer as a result of incomplete mTOR inhibition^39^. Although this dual inhibition of mTORC by SHK still needs more investigation, these data at least partly suggest SHK might directly target mTORC1/mTORC2.

Long-term exposure of the tumor to certain drugs can lead to drug resistance. Multi-drug combination is a promising approach to overcome drug resistance^40,41^. The effect of SHK combined with DOX was evaluated and we found that low dose SHK potentiated anti-proliferation activity of DOX in BL cells. The molecular mechanisms of this synergistic effect may be involved with enhancement of apoptosis. Furthermore, our findings *in vitro* have been recapitulated *in vivo* in a subcutaneous BL model, indicating that the modulation of C-MYC and PI3K/AKT/mTOR is, at least, one of the molecular mechanisms by which SHK may manifest its effect against BL.

In summary, our results indicate that cytotoxic effect mediated by SHK against BL attributes to caspase-dependent apoptosis. Inhibition of C-MYC and suppression of PI3K/AKT/mTOR activity play critical roles in SHK-induced apoptosis in BL both *in vitro* and *in vivo*. We also demonstrated that SHK has property to enhance chemotherapeutic sensitivity in BL cells. Altogether, these data suggest that SHK may be an encouraging chemotherapeutic agent in the clinical treatment of BL.

## Materials and Methods

### Cell lines and reagents

The Raji and Namalwa human Burkitt’s lymphoma cell lines (both of them are EBV-positive) were obtained from the American Type Culture Collection (Manassas, VA, USA). All cells were cultured in RPMI-1640 culture medium (Corning, NY, USA) supplemented with 10% fetal bovine serum (Gibco-RRL, Grand Island, NY, USA) at 37 °C in a humidified incubator with 5% CO_2_. Shikonin was purchased from Sigma- Aldrich (St Louis, MO, USA) and dissolved in dimethylsulfoxide (DMSO) at a concentration of 20 mM, then stored in the dark at −20 °C. Doxorubicin, ZVAD-FMK and C-MYC inhibitor 10058-F4 were purchased from Selleck Chemicals (Houston, TX, USA) and dissolved in DMSO at a concentration of 20 mM, then stored at −20 °C. Stock solutions of these agents were subsequently diluted with the serum-free RPMI-1640 medium before use. The final concentration of DMSO did not exceed 0.1% in all experiments.

### MTT

Cell viability was monitored by 3-(4,5-dimethylthiazol-2-thiazolyl)-2,5-diphenyltetrazolium bromide (MTT; Sigma) assay following the manufacturer’s instructions. Briefly, cells (2 × 10^4^ cells/well) were seeded in 96-well plates and treated with drugs for the indicated times. After incubation, 20 μl of MTT solution (5 mg/ml) was added to each well and then the plates were incubated at 4 °C for 3 h. The absorbance of the reaction was measured by a 96-well plate reader (Bio-Rad, Hercules, CA, USA) at 570 nm. IC50 values (half maximal inhibitory concentration) were calculated.

### Flow cytometric analysis

Annexin V and 7-AAD staining kit (Multi Sciences, Hangzhou, Zhejiang, China) was applied to quantify the rate of cell apoptosis. After treatment, cells (5 × 10^5^ cells) were collected and washed twice with cold phosphate-buffered saline (PBS). The cells were resuspended in 100 μl of 1x binding buffer and incubated at room temperature for 15 min with 5 μl Annexin V-APC and 10 μl 7-AAD in the dark, then 400 μL of 1x binding buffer was added to each tube. The stained cells were quantified by flow cytometer (D3130, ACEA Biosciences, China).

### Western blot analysis

Cells were treated for the indicated time in the presence or absence of drugs. The cells were then washed twice with PBS and lysed in cell lysis buffer (Cell Signaling Technology, Beverly, MA, USA) containing protease inhibitors. Protein concentrations of samples were determined using the BCA Protein Assay Kit (Sangon Biotech, Shanghai, China). Aliquots containing 30 μg proteins were separated on sodium dodecyl sulfate (SDS) -polyacrylamide gels containing 6–12% acrylamide gradients and then transferred to PVDF membranes (Millipore, Billerica, USA). The membranes were blocked for 2 h in Tris-buffered saline containing 0.1% Tween and 5% skim milk and then incubated with primary antibodies overnight at 4 °C. After washing three times, the membranes were incubated with anti-rabbit/mouse IgG antibody conjugated to horseradish peroxidase (Multi Sciences) for 2 h at room temperature. The results were visualized with the ECL detecting kit (Biological Industries, Cromwell, CT, USA). All primary antibodies were purchased from Cell Signaling Technology except C-MYC and p-mTOR (Ser2481) (Abcam, Cambridge, UK).

### Hoechst 33342 and propidium iodide (PI) staining

Hoechst 33342 (Beyotime Biotechnologies, Shanghai, China) and PI (Multi Sciences) staining were used to analyze the nuclear morphology. Cells (2 × 10^5^ cells) were treated with either DMSO (control) or shikonin at the indicated concentration for 6 h at 37 °C. After washed twice with PBS, the cells were incubated with Hoechst 33342 (1 mg/mL) and PI (5 mg/mL) at room temperature for 15 min and observed under fluorescence microscope (DFC450, Leica, Germany). Apoptotic cells were identified by morphologic changes in their nuclear assembly by observing chromatin condensation and fragment staining by Hoechst 33342 or both Hoechst 33342 and PI.

### Real-time PCR

Total RNA of BL cells were extracted using the TRIZOL Reagent (Invitrogen, CA, Carlsbad, USA) according to manufacturer’s instructions. Reverse-transcribed complementary DNA was synthesized with the Prime-Script® RT Reagent Kit (Takara, Tokyo, Japan). Amplification reactions were performed using SYBRP remix Ex Taq (Takara) in a 20 μL volume on a 96-well optical reaction plate in CFX96 Real-Time PCR Detection System (Bio-Rad). RT-PCR was performed with an initial denaturation at 95 °C for 5 min followed by 40 cycles including denaturing at 95 °C for 10 s, annealing at 60 °C for 20 s and extension at 70 °C for 10 min. Melting curves were computed after PCR product amplification. MiR-19a was normalized to U6. Comparative RT-PCR was conducted in triplicate, including no-template controls. The comparative cross-threshold (Ct) method was used to calculate relative expression. The primers for miR-19a and U6 were synthesized by RiboBio (Guangzhou, Guangdong, China).

### Combination index analysis

For evaluating the synergistic efficacies of shikonin and doxorubicin, the combination index (CI) isobologram method of Chou and Talalay was used. This commonly used analysis involves plotting concentration–effect curves for each agent and multiple diluted fixed-ratio combinations by using the median effect equation and the combination index equation. A value of CI less than, equal to or greater than 1 indicates synergism, additivity and antagonism, respectively. The synergy of Doxorubicin with shikonin was analyzed with the use of CalcuSyn software (Biosoft, Cambridge, UK).

### Establishment and treatment of Namalwa xenografts

All animal experiments were reviewed and approved by the Institutional Animal Care and Use Committee. All experiments *in vivo* were performed in accordance with relevant guidelines and regulations. Male non-obese diabetic/severe combined immunodeficient (NOD/SCID) mice (4 weeks old) were obtained from Shanghai Experimental Animal Center of the Chinese Academy of Sciences (Shanghai, China). For establishment of the xenograft, a mixture of 1 × 10^7^ Namalwa cells and PBS was injected subcutaneously in the right oxter of each mouse. The tumor volumes were determined by measuring length (L) and width (W), and calculating volume (V = 0.5 × L × W^2^) at the indicated time points. When each tumor volume reached about 100 mm^3^, the mice were randomly assigned to two groups (five mice per group): Vehicle, SHK (4 mg/kg). Mice were treated by intraperitoneal (i.p.) injection of 4 mg/kg SHK in fat emulsion every other day, or by i.p. injection of the same volume of the fat emulsion according to the same schedule. At the end of treatment, the mice were sacrificed, and the tumors were removed and weighed.

### Immunohistochemistry

The extracted tumor tissues were fixed in 10% formalin at room temperature, processed and embedded in paraffin. Paraffin-embedded tissues were sectioned (5 mm thick). For histopathology analysis, the paraffin sections of tumors were stained with HE. Tissue sections were primarily stained with indicated antibodies for immunohistochemical analysis. Then biotinylated secondary antibodies detected the signal with DAB. Images were acquired using a Nikon E300 fluorescence microscope equipped with a Nikon digital camera (400x).

### Statistical analysis

All data are presented as the mean ± SD for at least three independent experiments. Comparisons between two groups were performed using Student’s t-test and P < 0.05 was considered statistically significant.

### Data availability

Original data files are available upon a reasonable request.

## Electronic supplementary material

Electronic supplementary material
