# Supplementary material for: Shikonin exerts antitumor activity in Burkitt’s lymphoma by inhibiting C-MYC and PI3K/AKT/mTOR pathway and acts synergistically with doxorubicin
Source: Sci Rep. 2018 Feb 20;8:3317. doi: 10.1038/s41598-018-21570-z (PMC5820316; doi:10.1038/s41598-018-21570-z)

**Shikonin exerts** **antitumor activity in Burkitt’s lymphoma by inhibiting C-MYC and PI3K/AKT/mTOR pathway and acts synergistically with doxorubicin**

Fan Ni^1,2^, Xianbo Huang^4^, Zhenzhen Chen^4^, Wenbin Qian^4,*^, Xiangmin Tong^1,2,3,*^

Supplementary Figure 1: Full-length blots of Figure 2 in the main text.


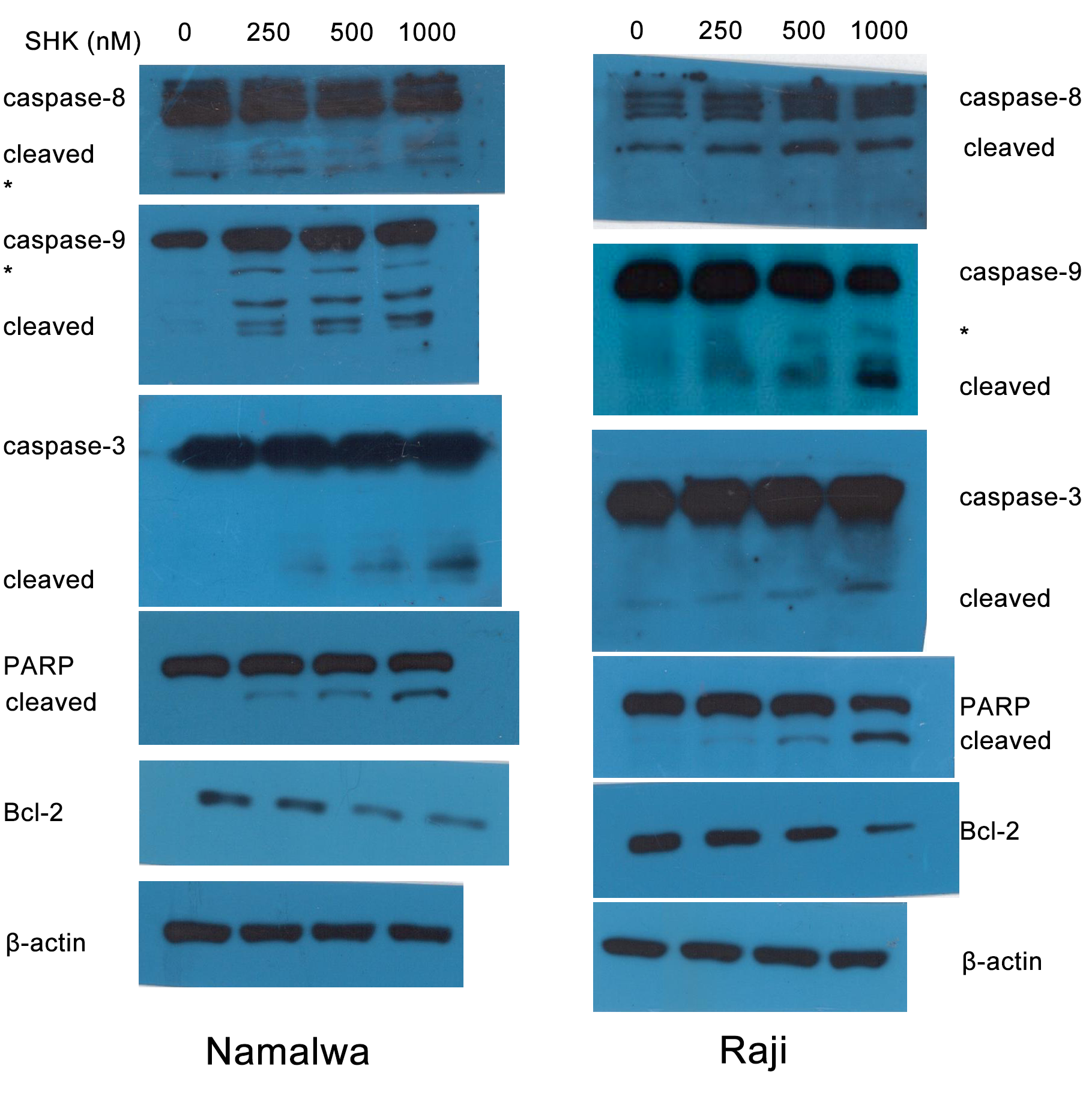


*unspecific band

Supplementary Figure 2: Full-length blots of Figure 3 in the main text.


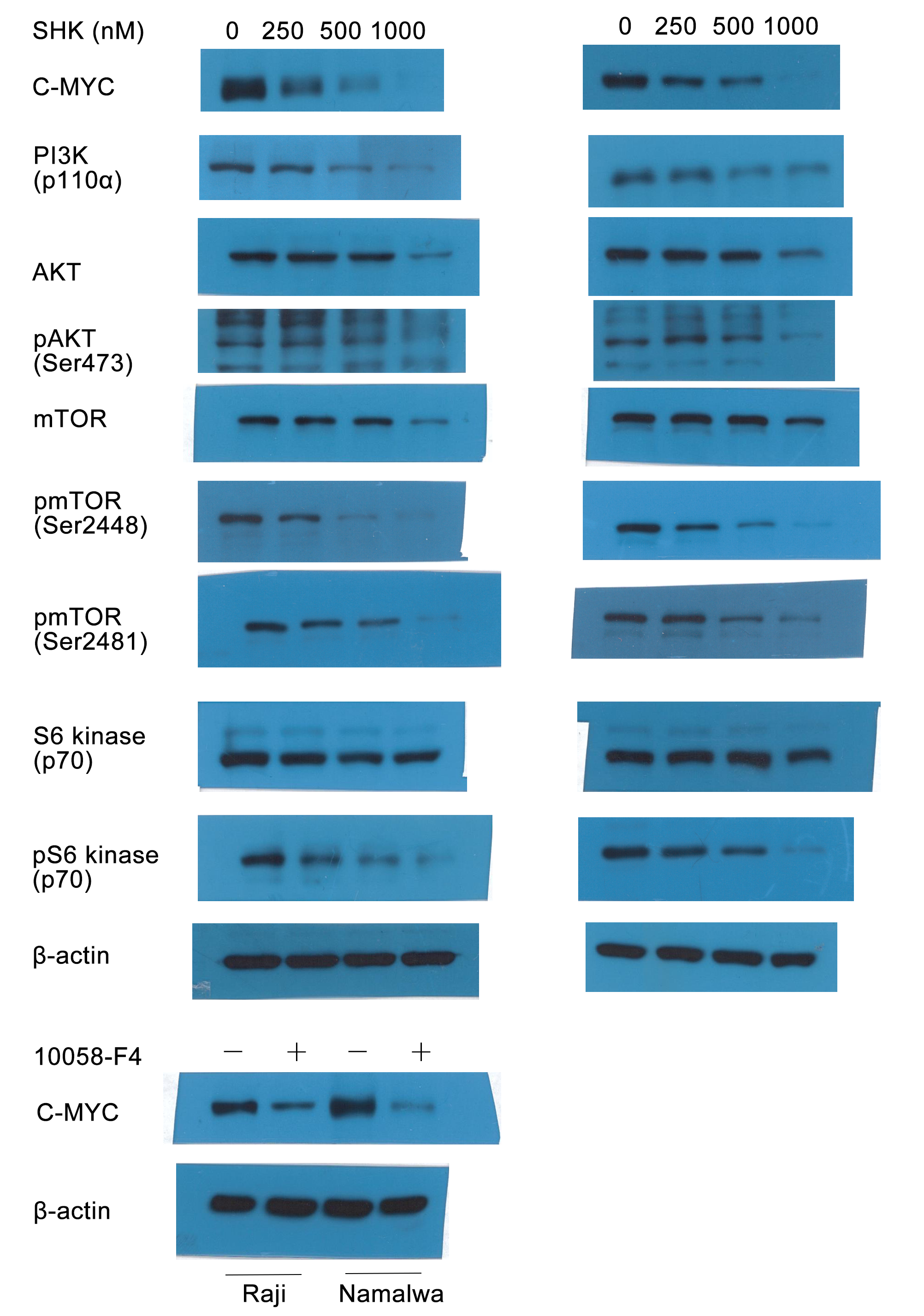
­­

Supplementary Figure 3: Full-length blots of Figure 4 in the main text.


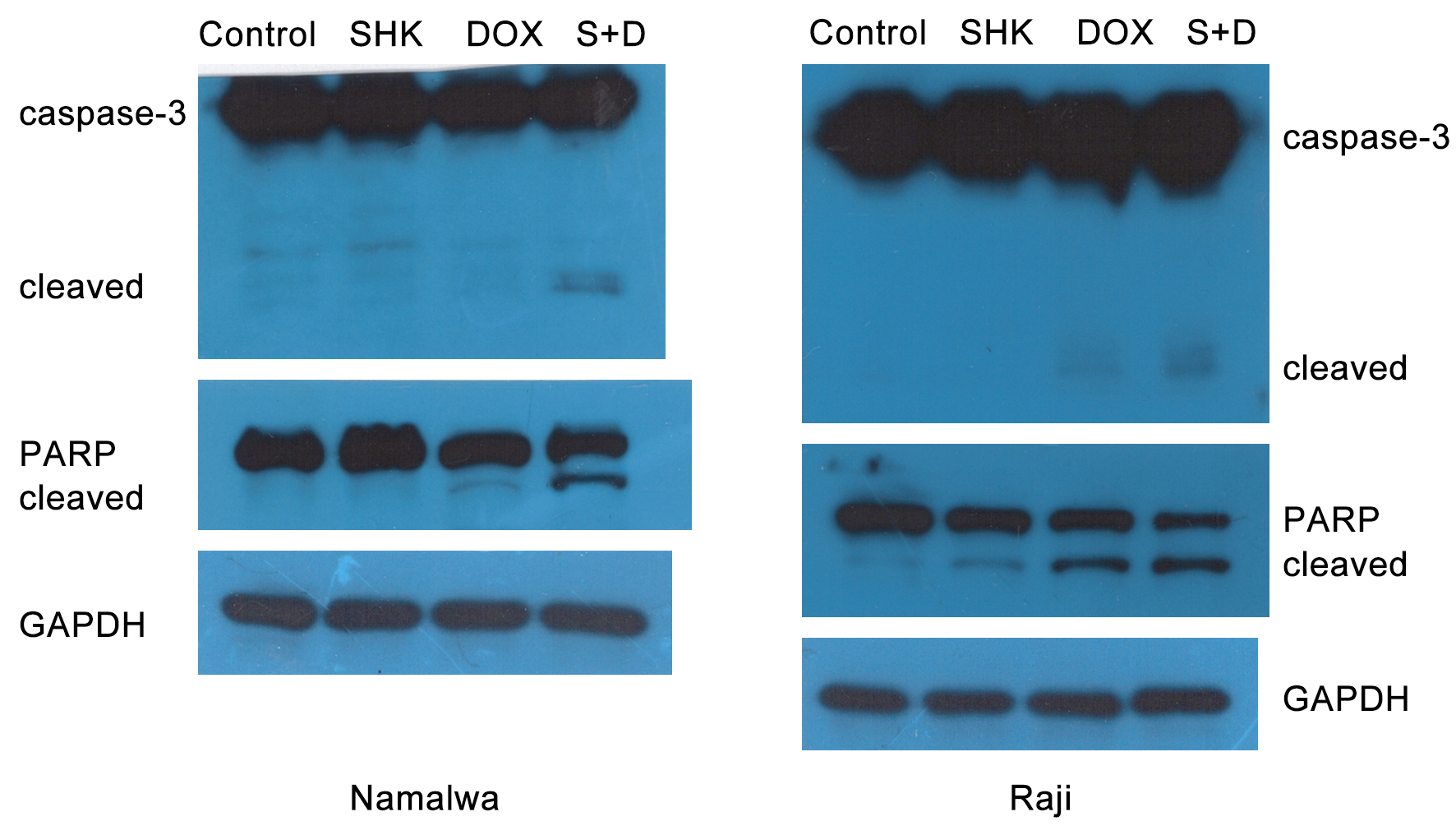


Supplementary Figure 4: Full-length blots of Figure 5 in the main text.


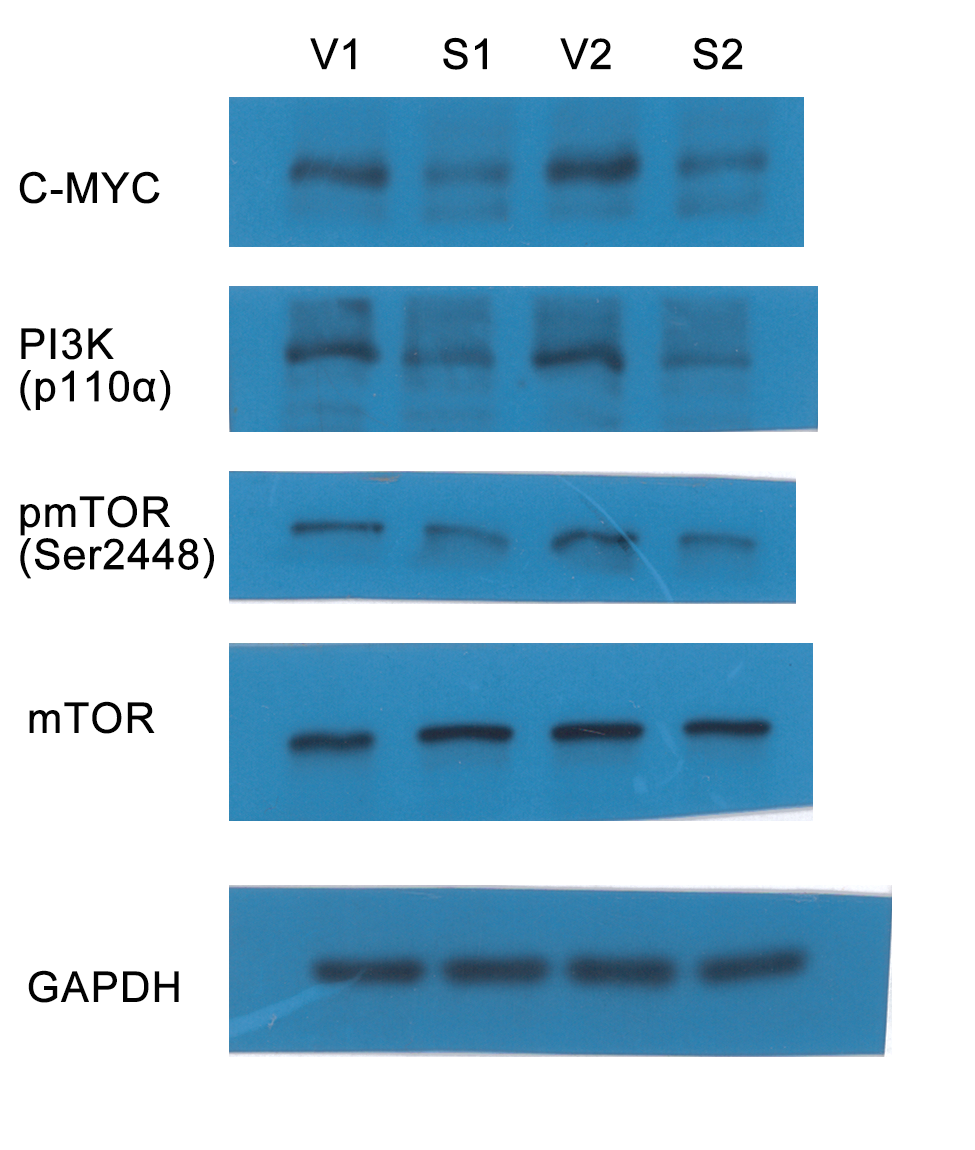

Supplement: Supplementary file 1 — Electronic supplementary material [file 41598_2018_21570_MOESM1_ESM.doc]
